# Widening Consumer Access to Medicines: A Comparison of Prescription to Non-Prescription Medicine Switch in Australia and New Zealand

**DOI:** 10.1371/journal.pone.0119011

**Published:** 2015-03-18

**Authors:** Natalie J. Gauld, Fiona S. Kelly, Lynne M. Emmerton, Stephen A. Buetow

**Affiliations:** 1 Department of General Practice and Primary Health Care, University of Auckland, Auckland, New Zealand; 2 School of Pharmacy, University of Auckland, Auckland, New Zealand; 3 Griffith Health Institute, Griffith University, Brisbane, Australia; 4 School of Pharmacy, Faculty of Health Sciences, Curtin University, Perth, Australia; Kilifi, KENYA

## Abstract

**Background:**

Despite similarities in health systems and Trans-Tasman Harmonization of medicines scheduling, New Zealand is more active than Australia in ‘switching’ (reclassifying) medicines from prescription to non-prescription.

**Objectives:**

To identify and compare enablers and barriers to switch in New Zealand and Australia.

**Methods:**

We conducted and analyzed 27 in-depth personal interviews with key participants in NZ and Australia and international participants previously located in Australia, and analyzed records of meetings considering switches (2000–2013). Analysis of both sets of data entailed a heuristic qualitative approach that embraced the lead researcher’s knowledge and experience.

**Results:**

The key themes identified were conservatism and political influences in Australia, and an open attitude, proactivity and flexibility in NZ. Pharmacist-only medicine schedules and individuals holding a progressive attitude were proposed to facilitate switch in both countries. A pharmacy retail group drove many switches in NZ (‘third-party switch’), unlike Australia. Barriers to switch in both countries included small market sizes, funding of prescription medicines and cost of doctor visits, and lack of market exclusivity. In Australia, advertising limitations for pharmacist-only medicines reportedly discouraged industry from submitting switch applications. Perceptions of pharmacy performance could help or hinder switches.

**Conclusion:**

Committee and regulator openness to switch, and confidence in pharmacy appear to influence consumer access to medicines. The pharmacist-only medicine schedule in Australasia and the rise of third-party switch and flexibility in switch in NZ could be considered elsewhere to enable switch.

## Introduction

Consumers can access different medicines without a prescription in Australia and New Zealand (NZ) respectively[1,2] despite similar health systems, populations,[3] and medicines schedules.[1] For example, inhaled salbutamol (for asthma) is available without a prescription in Australia but not NZ, and the opposite is true for some vaccines, and sumatriptan (for migraines).[1]

Prescription to non-prescription switch (reclassification or down-scheduling) may expose consumers to risks such as inappropriate use or delayed diagnosis.[4,5] However, benefits include convenient and timely access to medicines for consumers. Empowering patients to manage minor ailments and use doctors more efficiently can benefit the health system.[5] Such benefits may be significant, given the forecast increased pressure on health resources globally.[6]

Australia and NZ have long contributed to an international trend to switch medicines to non-prescription availability. While mid-2000s research found that Australia[7,8] and NZ[7] were relatively advanced in non-prescription availability of medicines, later research showed switch varied over time.[1]

Both countries have three non-prescription categories: general sales (unscheduled); pharmacy-only (Schedule 2 in Australia), and pharmacist-only (Schedule 3 in Australia).[9,10] In the United Kingdom (UK), Aronson[11] suggested that a pharmacist-only schedule with safeguards would increase control compared with pharmacy-only status, and therefore enable some switches.[11] Researching key opinion leaders’ attitudes, Achanta, *et al*.,[12] proposed that the United States (US) would benefit from a pharmacist-controlled class of medicines, but the US Government Accountability Office disagreed.[8] Research in two countries with pharmacist-only classifications could provide insight into the effect of a pharmacist-only category on widening consumer access to medicines through switch.

To our knowledge, neither Australia nor NZ has published a government position on switch. Advisory committees in both countries consider switch applications, and make recommendations, but the Minister of Health for NZ, and the Secretary to the Department of Health for Australia, or their respective delegates make the final decisions.

The National Drugs and Poisons Scheduling Committee (NDPSC) determined medicines and poisons scheduling in Australia until mid-2010.[1,13] It included ‘jurisdictional’ representatives (from two states, two territories and, for some of the time, NZ) and appointees (including consumer, industry and pharmacy representatives) and was chaired by the Commonwealth (Therapeutic Goods Administration; TGA). Decisions required a majority vote from jurisdictional representatives (i.e. those representing a jurisdiction such as a state or territory). For switch decisions the NDPSC considered: toxicity and safety; risks and benefits; potential hazards; the extent and patterns of use; dosage and formulation; need for access to a substance; potential for abuse; purposes for which a substance is to be used; and any other matters that the Committee considered necessary to protect public health.[14] These considerations were legislated.

From 2010, following a review, medicines and poisons scheduling in Australia were separated. The Advisory Committee on Medicines Scheduling (ACMS) advises on medicines scheduling only. Committee composition and switch considerations for the NDPSC and the ACMS are published elsewhere.[1] In brief, the ACMS comprises nine representatives of States and Territories and the Commonwealth government, and up to six independent expert members. For switch decisions, the ACMS considers: risks and benefits; purposes of use and extent of use; toxicity; dosage, formulation, labelling, packaging and presentation; potential for abuse; and any other matters that the Secretary considers necessary to protect public health.[15] These considerations are legislated.

Medicines policy in Australia around the time of the research had four central objectives: timely access to the medicines that Australians need, at an affordable cost; quality, safe and efficacious medicines; quality use of medicines; and maintaining a responsible and viable medicines industry.[16,17] The concept of access to medicines focuses on cost particularly. Scheduling is mentioned in helping address the risks of medicines. Self-help or self-selection of medicines receive minimal attention.

In NZ, the Medicines Classification Committee (MCC), a Ministerial advisory committee, recommends classifications of medicines as prescription medicines, pharmacist-only medicines, pharmacy-only medicines[18] or unscheduled medicines. Medsafe, NZ’s regulator of medicines and medical devices, administers this committee. The MCC comprises six members, two each nominated by the Ministry of Health (including the committee Chair), the Pharmaceutical Society, and Medical Association. Recommendations made by this committee are considered by the Minister of Health’s Delegate, along with advice from Medsafe. The Delegate may support the committee’s recommendation, or accept alternate advice from Medsafe. Following publication of the minutes, the decision will be gazetted unless there are valid objections. At the time of the research, the MCC considered the following when reclassifying medicines: consumer convenience; potency (i.e. effectiveness); current availability, therapeutic index; toxicity; abuse potential; inappropriate use (relevant to the condition being treated); precautions; and communal harm.[1]

Medicines policy in NZ around the time of the research aimed to deliver to New Zealanders quality, safe and effective medicines; access to medicines regardless of the ability to pay and within government funding; and optimal use of medicines resulting in optimal outcomes.[19] This policy made no mention of self-care, non-prescription medicines or switch. The Ministry of Health had a “better, sooner, more convenient” strategy for moving services from hospital into primary care, and try to keep people healthy in the community.[20]

Since the 1990s, NZ and Australia have been attempting to harmonize their scheduling of medicines, a process called Trans-Tasman Harmonization (TTH). Where the scheduling differs for a medicine, the intent has been to harmonize to the least restrictive schedule while considering public health and safety issues and/or jurisdictional needs.[21] Described in 2003 as a ground-breaking event,[22] to our knowledge such harmonization between two separate countries remains unique.

No published literature investigates the TTH scheduling initiative, and other research investigating effects on switch have often focused on a limited range of factors[7,8,12,23] or a single medicine.[24,25] Trans-Tasman analysis provides a unique opportunity to understand influences on switch in similar countries over time, and reflect on international harmonization policy. This paper therefore compares barriers and enablers to prescription to non-prescription switch in Australia *versus* neighboring NZ.

## Methods

The University of Auckland Human Participants Ethics Committee provided ethical approval (2008/304) for this study. A modified form of qualitative heuristic research was used. [26] It embraced the personal experience, knowledge and active input of the lead researcher (NG, a doctoral student during the research) who has a history of intense involvement and interest in the topic. Similar to the insider (emic) approach typical of much ethnographic case study research,[26] the heuristic approach promised to deepen our understanding of the nature and meaning of her lived experience of the study phenomenon. Table 1 summarizes how she modified for this study the heuristic approach described by Moustakas.[26,27]

**Table 1:** A comparison of the heuristic approach used versus the standard heuristic approach[26,27].

	Standard heuristic approach	The heuristic approach we used
Closeness to data	Connectedness and relationship	Connectedness and relationship
What is described	Portrays meanings and personal significance	Portrays meanings but not personal significance
Method	Extended interviews with up to 10–15 participants providing in-depth information stopping when they reach a natural close	A mix of extended interviews stopping when they reach a natural close, and shorter interviews. Supplementary document analysis.
Analytical processes	Creative synthesis including the researcher’s intuition and inferences	A mix of distillation and creative synthesis including the researcher’s intuition and inferences
Reporting	Individuals are portrayed as whole persons	Individuals have reduced visibility owing to confidentiality and the large sample size

At the outset of the study, NG believed that, on balance, switch with relative safety is desirable, but was mindful of the need to be constantly reflexive around how this position impacted on this research. NG was, at that time, a member of NZ’s MCC (2004–2009). She had supplied switched medicines as a pharmacist, had researched switched medicines, and had observed an Australian National Drugs and Poisons Schedule Committee (NDPSC) meeting (February 2007), and prepared switch applications for NZ (2010–2014). Effectively becoming a co-participant in this study, she drew upon her professional experience to create a milieu conducive to eliciting rich information. To this end she also shared her professional experience toward the end of interviews. She sought to be reflexive by triangulating field notes, including reflective notes, with data collected in both countries from documents and interviews. She used introspection to examine her thoughts and feelings, and subjected these to further scrutiny through skeptical peer review. Participants checked research outputs to enhance the trustworthiness of the research. NG was an experienced interviewer. The other authors were supervisors or an advisor on this work, and informed the study design, monitored its implementation and provided skeptical peer review of analysis and reporting. FK and LE are academic pharmacists who have worked in pharmacy practice research (including switch-related projects) in Australia and NZ. SB is a social scientist working in primary health care.

The flow chart in S1 Appendix outlines the method used. We sought to maximize variation in the sample, and identify themes that cut across this variation.[26] Key participants were purposively selected therefore from diverse groups, specifically regulatory authorities, pharmacy organizations, pharmaceutical industry, doctors’ groups, consumers’ groups, academia, and the committees considering switches of medicines, as agreed by the research team. Most participants were selected for their knowledge of or involvement in switch according to pre-existing knowledge held by NG, and sometimes referred by other participants (snow-balling), with assent from the research team. Often only one person in an organization was responsible for switch. NG sought consciously to include people known or likely to hold views contrary to her own. The sample therefore was biased towards efficiently including participants who, within particular occupational niches, had the most experience of switch.[28] As Morse explains, “the sample is biased; it *must* be biased”.[p734]

Initial contact occurred by telephone, then email or email alone for persons already known. Where no such key person was known, the organization was telephoned with a request to interview the most appropriate spokesperson about switching medicines from prescription to non-prescription, followed by email. For pharmacy organizations, typically the employee who was most involved in switch submissions was interviewed as well as a Board member to gain both a hands-on view and a broader understanding. Data was included from two participants who were interviewed for the international part of this study and had experience of switch in Australia. Following informed consent (written and/or oral), NG conducted semi-structured interviews face-to-face or by telephone from late 2009 to early 2012, at a mutually acceptable time and location.

NG had previously communicated with 18 participants. Four participants had long-standing relationships with NG. All participants were informed that the research was doctoral research exploring reasons for variation in switch in different countries. Most participants were aware of NG’s role on the MCC in NZ.

The questions were iterative, evolving with successive interviews on the basis of emergent knowledge, depending on the participant’s role and on comments raised in the interview. Table 2 reports the general topics covered. Field notes were taken. The interviews were audio-recorded and transcribed verbatim. The transcription was offered to participants to review.

**Table 2:** Usual lines of inquiry.

• The participant’s role and/or his/her organization’s role in medicine switch
• How he/she considered their country compared to other developed countries in prescription to non-prescription switch, and why
• Barriers to switch in their country
• Enablers to switch in their country
• Why NZ and Australia differ in switch
• His/her opinion on their country’s schedules with respect to their effect on switch
• His/her opinion on influence of decisions elsewhere on their country
• His/her opinion on market exclusivity for switched medicines
• His/her opinion on advertising for switched medicines (Australia)
• His/her opinions on having different stakeholders work together in switch

NG conducted analysis and reporting with input from the co-authors (see S1 Appendix). Interview transcripts were carefully read and reread. Concepts identified in the transcripts were labelled (coded). Codes were grouped thematically in context, using the qualitative software package, NVIVO 9 for data management. This iterative process segmented themes by country, or commentary on TTH. Then broad areas and themes that emerged from the data before or during coding provided the next level of coding, with further levels as appropriate, e.g. for commentary about specific medicine switches. Many interview segments fitted into general topics and themes. After preparing a full report for each country and comparing the two countries, transcripts were re-read to ensure quotes were used in context and the overall feel of interviews was reflected. Data saturation was not sought owing to the limited number of persons available with switch experience. A NZ participant reviewed the NZ report, an Australian participant reviewed the Australian report, and a participant with long-standing experience in switch in both countries reviewed both reports. Minor modifications resulted from review.

NZ documents analyzed included MCC meeting minutes which were obtained from the Medsafe website (1999–2012) and from Medsafe (1990–1999). Australian documents analyzed included records of NDPSC meetings (2000-June 2010) and ACMS meetings and delegate decisions (August 2010-December 2012). The NDPSC and ACMS documents were sourced from the website of the Therapeutic Goods Administration (TGA; Australia’s medicines regulatory agency). Prescription to non-prescription switch decisions and their reasons were summarized into tables of decisions, and summaries of reasons, coded by medicine. All meeting records were reread and checked against the tables and summaries to ensure accuracy. For medicines that emerged strongly in the interviews, specifically sumatriptan, chloramphenicol, orlistat, potassium chloride, calcipotriol and oseltamivir, relevant meeting records were extracted verbatim, coded by medicine and analyzed with interview data to include in the country reports. One case study has been published elsewhere.[29]. Other documents were obtained as needed for analysis and reporting. For Australia, these documents comprised: media reports of the orlistat advertising and mystery shopping, and the Pan Pharmaceuticals recall; the coroner’s report of the potassium poisoning case; and media statements on switch from medical organizations. For NZ, the documents included switch applications.

## Results

Analyzed interviews comprised 10 NZ, 15 Australian, and two international participants. Participants were from the medicines regulator (n = 3); pharmaceutical industry (n = 8); pharmacy organizations (n = 7); medical organizations (n = 3); pharmacy academia (n = 3); and a consumer organization (n = 2). A further participant was there solely in their committee capacity. Some participants had experiences of switch in both countries, and many had committee experience: from the NDPSC (n = 6); ACMS (n = 1); and/or MCC (n = 2). Interviews were conducted for 25 minutes to 2.5 hours, with most taking around one hour. One NZ industry person declined to be interviewed. A NZ pharmacy organization interview was discarded after recorder failure on the phone interview.

### Overall themes

Dominant themes from the interviews were risk averseness or political conservatism in Australia, and an open, flexible environment in NZ. The Australian environment for switch changed, from relatively progressive in switch in the early 2000s to conservative around 2006–2007 onwards. NZ participants also spoke of their country being used to change or where change could happen easily.

### Enablers and barriers

**Fig 1:**
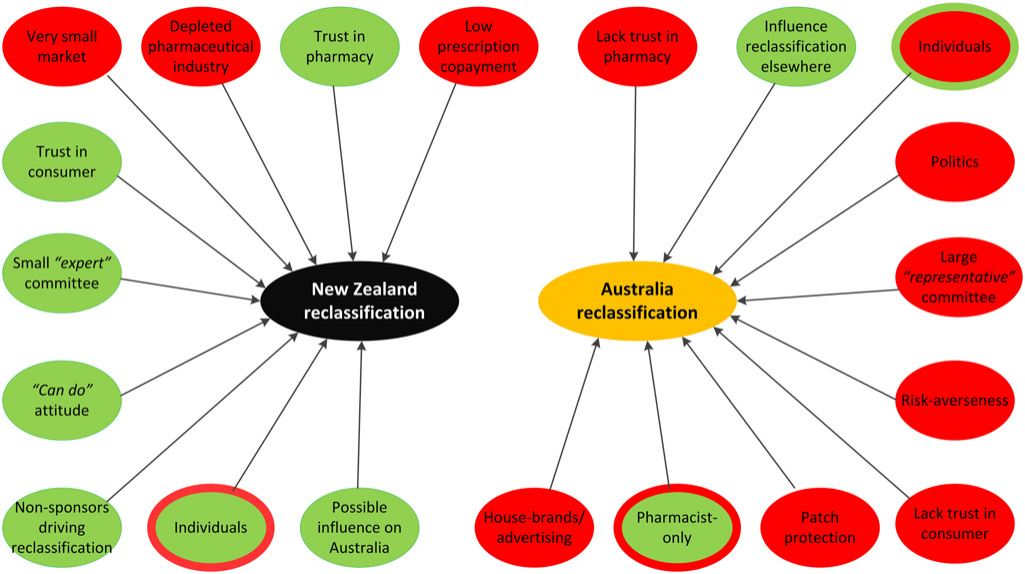
Key reasons for Trans-Tasman variation.

Enablers and barriers differed greatly between the two countries, with NZ data demonstrating more enablers (Fig. 1 and Table 3) and Australia revealing more barriers (Fig. 1 and Table 4). Similar enablers or barriers between the countries commonly appeared stronger in one country. For example, small population size as a barrier received several mentions in Australia, but emerged strongly for the less populated NZ. A barrier could also be an enabler, e.g. small population size limited potential sales in NZ, but helped the open, flexible, proactive attitude and appeared to limit political pressure.

**Table 3:** Enablers in Australia and New Zealand for switches (based on interview data).

Australia	New Zealand
Pharmacist-only schedule	Pharmacist-only schedule
Low cost of applications	Open to different ideas
Confidence in pharmacy	Industry confidence in committee
Individuals (less so recently)	Confidence in regulator
	Potential to influence switch in Australia
	Confidence in pharmacy
	Confidence in consumers
	Working with stakeholders
	Low cost of applications
	Can advertise OTCs
	Individuals
	Small country

**Table 4:** Barriers in Australia and New Zealand for switches (based on interview data).

Australia	New Zealand
Advertising restrictions	Small population
Risk averse committee	Negative prescription environment
Politics	Immediate generic entry
Immediate generic entry	Low co-payment on prescriptions
Patch protection	Lack of proactivity at the pharmacy level
Concerns about pharmacy	Patch protection
Pharmacy house-brands	Larger, certain prescription environment
Pharmacy organization conservatism	Limited NZ presence of companies
Small population	
Inability to see regulator	
Lack of pharmacy proactivity	

### Themes in medicines switch in NZ

#### Pragmatic, proactive, open attitude and change

Many participants in NZ alluded to an open attitude to switch and flexibility. Examples of this attitude from the committee (based on interviews, meeting minutes and the lead researcher’s experience) included proactively suggesting switch candidates (evident in meeting minutes), and being open to switches that were firsts for the developed world. The regulator allowed an exemption to prescription instead of the pharmacist-only category to overcome the need for labelling changes, and allowed a switch with mandatory training.

*“I think the Chair and the Chair’s willingness to engage with industry and say ‘come on let’s put in a submission’, get in, advocate for change, you know it’s risky but it’s required in the NZ environment because otherwise we’d have nothing to look at, basically.”*

*Regulator participant*

Many participants (including some from Australia) believed that the pragmatic approach by the MCC Chair enabled switch.

The open attitude has sometimes been less apparent, with a seemingly conservative stance in the late 1990s to 2003. Meeting minutes revealed reverse switches (returning to prescription medicine) under TTH for which safety concerns were not apparent, e.g. with oxybutynin (for incontinence). In 2002, NZ’s MCC rejected switching a topical, moderate-potency corticosteroid (for dermatitis and eczema) despite its switch approvals in the UK and Australia. The MCC later (2005) recommended the switch be approved.

**Fig 2:**
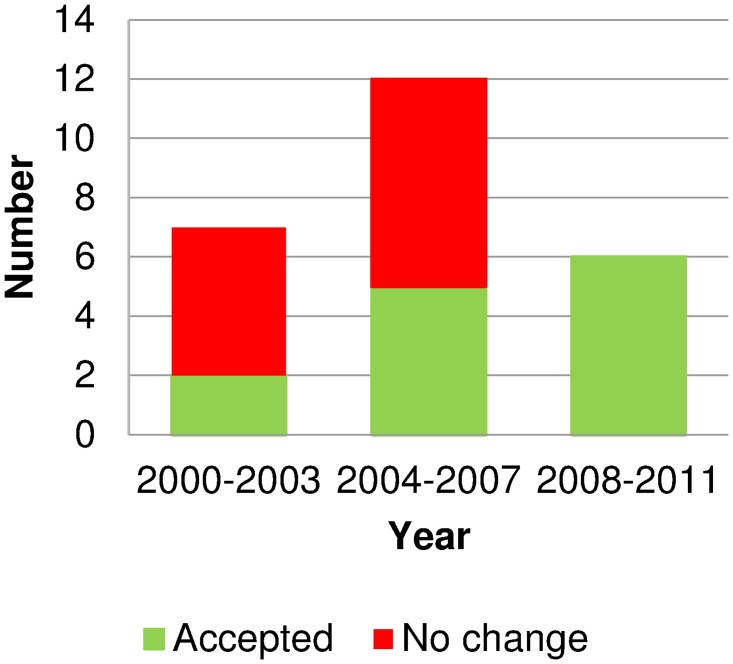
Outcomes of ‘progressive’ switches considered in New Zealand in 2000–2011. Source: Medicines Classification Meeting minutes. Medicines were counted once per period, regardless of the numbers of considerations for each medicine. The Medicines Classification Committee had three members change in 2004.

Some switches have required many meetings to gain approval, despite switching elsewhere (e.g. chloramphenicol for eye infections and omeprazole for heartburn). Others were not approved (e.g. simvastatin for prevention of cardiovascular events). These findings signify the influence of the overall committee and attitudes of individual members. Fig. 2 shows the MCC recommendations for progressive switches from 2000 to 2011 in four-year increments. Committee approvals were more common from 2004, when the committee membership changed significantly.

*“…you had to get the right GPs in the committee to make this happen, they had to be open-minded enough to say that there are lots of different ways of treating patients, and that doctors don’t have to be involved in every consultation…”*

*Regulator participant*

Proactivity was seen in non-sponsors driving switches, strongly contributing to NZ’s recent progress. Meeting minutes show that the regulatory authority drove the emergency hormonal contraceptive switch in 2001, and the committee drove chloramphenicol in 2006–2009, with input from the Pharmaceutical Society including developing training for both switches. From 2010 to 2013, a pharmacy retail group, Pharmacybrands (now Green Cross Health), applied to switch seven medicines, despite not sponsoring any. NG believes that NZ’s flexible, open approach was integral to achieving these switches, as discussed elsewhere for topical calcipotriol.[29]

An element of change was evident: NZ as a country was open to change, and, as a small country, NZ could change quickly. For example, the MCC (June 2006 minutes) noted a particular switch “*could easily be reversed*” if necessary, helping the switch to be approved. The committee continued to evolve (as shown in minutes and from an interview), and one participant suggested it had become more evidence-based in its decision-making. Two participants commented that NZ generally sometimes made changes too quickly, but did not relate this to any specific switches.

#### Trust

Interviews, minutes and the lead author’s experience indicate that committee and regulator trust in pharmacy and the consumer enabled switches in NZ. The regulator participant reported increasing committee trust in pharmacy, helped by pharmacy’s performance after switching oseltamivir (for influenza):

*“Pharmacy policed their profession very, very effectively and self-managed their profession very, very effectively, in a way that certainly makes me more confident in other things can go down in the same way….”*

The Pharmaceutical Society regularly informed pharmacy of switches and their responsibilities around providing switched medicines, and developed training to support some switches.

The MCC meeting minutes for trimethoprim in 2012 confirm the confidence in pharmacy, with the MCC removing the proposed need for a previous doctor’s diagnosis stating: “*with the correct training there was no reason why pharmacists could not manage patients who present for the first time with a urinary tract infection*”. Furthermore the meeting minutes reported “*… that pharmacist sale of trimethoprim may be more in line with best practice than the prescribing habits of general practitioners*.”

Pharmacy and non-pharmacy participants were generally positive about pharmacy’s abilities, citing improved undergraduate training, and re-professionalization of pharmacists. However, most participants who were pharmacists reported occasional poor practice, publicized in covert shopper studies. Both medical participants worried about pharmacy assistant training, and one lamented that pharmacists are less trained in diagnosis than doctors.

Recent switches have seen an increase in the use of screening tools and rise of mandatory training. For example, only pharmacists who have completed approved training can supply trimethoprim or administer vaccinations without prescription, using screening tools. Such tools may help generate trust in pharmacy to supply the medicines correctly.

Industry participants trusted the MCC, believing its decisions to be evidence-based.

#### Global and regional influences

An industry participant suggested NZ could be a test market, noting that “*if it tanks*, *it doesn’t matter… we haven’t lost anything*. *If it goes great*, *we’ve got experience then to provide to the Europeans*, *the FDA…*” NZ potentially influences Australia through TTH, with industry sometimes timing the NZ review before the Australian review.

Australia affected switch in NZ. Some switches occurred in Australia before NZ (e.g. orlistat for weight loss and fluconazole for vaginal thrush). Reverse switches (up-scheduling) occurred in NZ to harmonize with Australia, particularly around 1999–2000 (e.g. oxybutynin for urinary incontinence and colestyramine for cholesterol lowering). Influence from the UK (particularly), but also the US, Canada and Denmark was evident.

#### Financial influences

The financial theme arose in all interviews (Table 5). Document analysis shows that global pharmaceutical companies (the main drivers of switch internationally) have not driven ‘progressive’ switches in NZ since 2009. ‘Progressive’ switches are prescription to non-prescription switches judged to provide consumer benefit over existing non-prescription medicines. The small market and medicine funding policy reportedly make NZ unattractive for pharmaceutical companies, resulting in little or no NZ presence, and reducing the ability and desire to drive switch.

**Table 5:** Financial influences on switch in New Zealand.

Enablers	Impediments
Pharmacy dispensary funding squeeze increases interest in retail	Low prescription prices reduces company engagement
Part capitation-funding for doctors may reduce patch protection	Low co-payment incentivizes consumers to have medicines prescribed
No application fee for switch	Low volume reduces viability
Special studies not required	Consumers expect low-cost medicines
Switches to general sales may make pharmacy want more switches from prescription to replace lost income	Medsafe has insufficient resource to drive switches (as done previously)
	Immediate generic entry (if off patent)
	Pharmacy concentrating on dispensary
	Some patch protection (doctors and pharmacy)
	Pharmaceutical companies concentrate on the larger, more certain prescription market

Industry, pharmacy and medical participants suggested that subsidized health care limited self-medication. A medical participant considered subsidized care protected consumers: “*…there’s a connection between being uneducated and their financial set up*, *so they’re less likely to be able to buy OTC [over-the-counter] medication…*. *that takes some of the fear away*, *because it’s cheaper for them to come to me*.” Subsidized care was highlighted as a safety net in the switch application for topical calcipotriol (for psoriasis), incentivizing patients using large quantities to seek a doctor’s prescription.

The transparency of switch and resulting potential for immediate generic competition reportedly discouraged companies from driving switches. Industry participants desired market exclusivity (an exclusive period selling the switched medicine) to encourage switches, given the cost and effort of driving the switch and training. However, financial pressures on pharmacy were thought to encourage their interest in switch.

#### The pharmacist-only schedule

Most participants considered the pharmacist-only category enabled switches, as requiring health professional contact was believed to reassure the committee.

Many recent switches used an exemption to prescription availability through a pharmacist under strict criteria. Such criteria have sometimes included mandatory pharmacist training, used with the emergency hormonal contraceptive, vaccinations and trimethoprim (for urinary tract infections). This model tightens the pharmacist-only availability, minimizing risk, and/or precludes the need for non-prescription labelling.

### Australia

Australia progressed in the early 2000s with multiple innovative switches. The Chair and other committee members were reportedly open to switch, and industry personnel could discuss planned switches with jurisdictional members.

**Fig 3:**
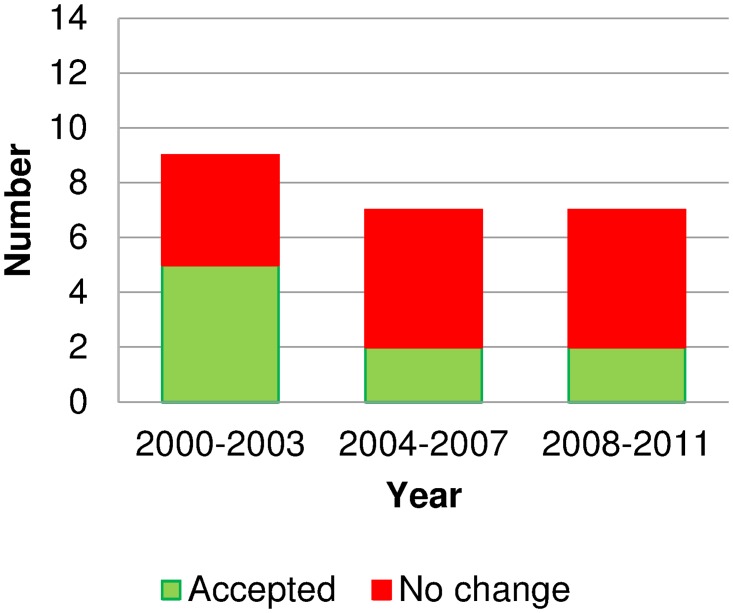
Outcomes of ‘progressive’ switches considered in Australia in 2000–2011. Source: Therapeutic Goods Authority records. Medicines were counted once per period, regardless of the numbers of considerations for each medicine. Tranexamic acid reclassified in 2000 but was reversed in 2007 when no non-prescription product had been marketed.

Fig. 3 provides the level of approvals (or recommended approvals after mid-2010) versus rejections for progressive switches in Australia, showing most progressive switch attempts since 2006 have been rejected. Participants suggested that increased conservatism in the NDPSC around 2005/2006 had multiple causes: changes of committee members; an international increase in risk-averseness affecting the TGA; increasingly complex switches; and local events such as orlistat advertising (see below). Industry personnel could no longer discuss planned switches with jurisdictional members. The key themes from interviews were risk-averseness and distrust, financial influences, global influences, pharmacy, and the effect of individuals.

#### Risk-averseness or conservatism and perceived lack of trust

The related themes of risk-averseness or conservatism and lack of trust arose in most interviews. Industry, pharmacy, and committee member participants commonly described the committee and regulator as risk-averse or conservative. In contrast, the medical and consumer participants did not suggest committee conservatism. The medical participant worried about effects of some switches that had occurred. Perceived risk-averseness in switch and advertising decisions discouraged industry from applying for switches.

Various participants suggested political pressure encouraged the risk-averseness.

*“…their life is to avoid risk…. We’ve got to keep our Minister happy and all these other stakeholders…”*

*Pharmacy participant*

Many participants, including committee members, suggested that jurisdictional members (considered inherently conservative) impeded switch, sometimes reportedly because of instructions from above.

*“…because of … the committee structure of the NDPSC and the voting arrangements specifically, it is ultimately about a series of government or bureaucracy agendas… versus a true evidence-based assessment.”*

*Industry participant*

Although predominantly viewed negatively, such conservatism was sometimes considered appropriate, usually by committee members. Several participants (including industry) suggested the risk-averseness was a push-back response to industry’s push.

*“…it’s better, in this area of healthcare, to tread warily. And in fact the resistance of those state jurisdictions to that change is almost the counterbalance to the arguments made by the sponsors for the change…”*

*Committee member*

The instructions from above were not always conservative. A former NDPSC member spoke of the progressive effect on switch from a “*liberal*” chief pharmacist who “*certainly gave riding instructions to his people that did represent [his state]*.”

Participants from industry, pharmacy and medicine described pharmacists as conservative, cautious or under-confident with switched medicines. Some participants reported that this limited sales.

*“…if you don’t prepare the profession, the profession clinically is very, very conservative, they’re scared, so they’re not going to touch anything like that.”*

*Pharmacy academic*

Although industry was not described as risk-averse or conservative, one participant commented that fear of medical backlash prevented oral contraceptives switching, and that potential pharmacy backlash delayed nicotine replacement moving into supermarkets. Pharmacy organizations reportedly opposed advertising of most pharmacist-only medicines and some prescription to pharmacist-only switches.

Interviews indicated the committee lacked trust in consumers, pharmacy and industry. One participant reported that Australia and NZ viewed the consumer differently. He reported that the MCC considers “*what harm can come to a reasonable NZ consumer if they buy a pack of this medicine from a pharmacy or a supermarket*, *and they read the label?*” He considered that the Australian committee starting position is: “*could the consumer get into harm if we lock them in the room with a bucketful of this medicine in the dark?*”

Some committee participants suggested that the committee lacked confidence in pharmacy, citing concerns about variable pharmacy behavior, and pharmacy assistants supplying medicines rather than pharmacists who were often busy.

*“…it’s almost as if that they allow it to go S3 [pharmacist-only], they’re sort of happy well that if the pharmacist doesn’t do his job properly, it probably wouldn’t be too bad.”*

*Committee member*

Meeting records for oseltamivir (for influenza) indicated concerns about misdiagnosis with pharmacist-supply. Meeting records for orlistat showed that the approval for advertising of this pharmacist-only medicine was revoked with the rationale including that advertising to consumers “*increased pressure on pharmacists to provide orlistat to consumers*. *This in turn had the potential to result in inappropriate patterns of use…”*

Various participants suggested that distrust in consumers and/or pharmacists arose from certain events (see “political influence” below), including mystery shopping in pharmacies; a move towards discounting medicines (potentially limiting time for consultations); personal experience of the committee members or their families when shopping in pharmacies; a desire to avoid risk (see “political influence”); and jurisdictional members’ awareness (through their work) of inappropriate pharmacist behavior. However, the recommendation to reclassify chloramphenicol (for red eye) in 2009 showed confidence in pharmacy with the NDPSC records reporting that several committee members contended that the “*rate of misdiagnosis was unlikely to differ significantly whether it was a pharmacist or a GP doing the diagnosis*”.

#### Political influence

The recent reported risk-aversion is not entirely unexpected, given media-fuelled events cited by participants, including a major recall instigation, and a child’s death through potassium chloride overdose.

*“… we live in a political climate…. And I think where there’s safety issues, people are more informed, and they’re more likely to take those issues public. And governments I guess are a little bit concerned about that… But I don’t necessarily see that as a bad thing.”*

*De-identified quote*

**Fig 4:**
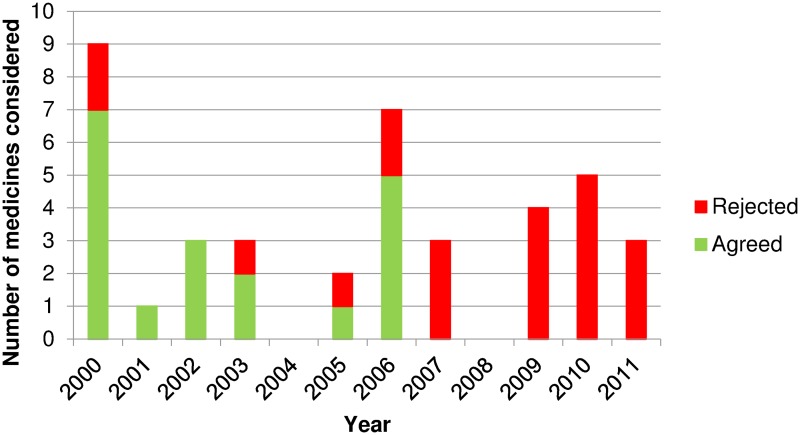
Advertising decisions in Australia for pharmacist-only medicines 2000–2011. **Source**: Therapeutic Goods Authority records. Some medicines appear multiple times, e.g. proton pump inhibitors from 2009.

Orlistat (for weight loss) became the watershed moment in advertising approvals. Orlistat advertising during a television program aimed at teenagers, stimulated an official advertising complaint (partly upheld)[30] and media interest. A subsequent Australian Consumer Association covert shopping study found that 24 of 30 pharmacies sold orlistat to a woman with a lower Body Mass Index than the licensed indication.[31] NDPSC records noted that submissions from professional organizations, pharmacy boards and consumer organizations “*strongly argued*” against advertising because of inappropriate pressure on pharmacists and potential to mislead consumers.[32] The NDPSC considered consumer advertising of orlistat could “*…lead to inappropriate patterns of use…*”, removing the advertising approval to “*…protect public health*”. Since 2007, all applications to advertise pharmacist-only medicines have been rejected (Fig. 4) noting “*no public benefit*”.

*“…the consumer people jumped on them from a great height and it became a political thing rather than anything that was based on evidence…”*

*Pharmacy participant*

Most strongly voiced about the committee, politics also arose from medical and pharmacy organizations, and was used by consumer organizations to highlight pharmacy or advertising concerns. The medical participant interviewed considered that the emergency contraceptive pill and vaginal antifungals (both long-standing switches) should be prescription-only, given that doctors were usually accessible for urgent matters. Conversely, a pharmacy participant considered doctors very inaccessible.

#### Committee issues

Committee conservatism is considered above. Various participants indicated that NDPSC decision making was sometimes not evidence-based, even with the usual independent evaluation. Participants suggested a large workload, inadequate preparation, the regulator’s influence, and nature of the membership affected decisions.

*“… it seems that no matter what the so-called experts think, it’s all up to the bureaucratic representatives, and I’m not too sure whether that’s good…”*

*Pharmacy participant and former committee member*

*“[NDPSC members are] also State employees, so they’ve got a direct line into the State government, which means they’ve also got that downwards pressure, that they’re not there as experts, they’re there as representatives.”*

*Committee member*

Some participants proposed that coal-face experience on the NDPSC with a community pharmacist and practicing general practitioner would be helpful. Several committee participants reported emotive decisions, and sudden turns in meetings.

*“…[committee members] wait for one to take the lead … and the rest would just rush in to support.”*

*Committee member*

*“…you take one committee, and they’ll make one decision on one day, and you bring them back two months later, and they might come to a different decision … it just depends on who’s most persuasive on the day.”*

*Committee member*

Various participants disagreed with the rejection of sumatriptan (for migraines), and one reported that the sponsor would address the committee’s issues and then new ones would arise, as supported by meeting record analysis across four meetings. A committee participant noted committee concerns about very rare events with sumatriptan, and suggested that trust was an issue:

*“[The committee] took the view that people with severe headache would go to the pharmacy and would buy sumatriptan, or pharmacists would want to sell them sumatriptan, so we’re in a trust question again… and then a complete lack of trust in the Australian consumer behaving reasonably.”*

The change in committee from NDPSC to ACMS reduced the committee size and workload, and changed the final vote from jurisdictional members only to all committee members, but jurisdictional representatives remain the majority of the ACMS. Subsequent switch and advertising decisions (Figs. 3 and 4) suggests conservatism remains. A participant added that government health policy initiatives, such as taking more personal responsibility for health, had yet to translate into self-medication, and no specific government policy supports switch.

#### Financial effects

Pharmaceutical companies were discouraged by financial factors, particularly advertising limitations, lack of market exclusivity, and to a lesser degree a small market. Industry was frustrated at the “*product graveyard*” of switch without advertising (“*S3 non-advertisable*”). While about one-third of participants expressed concern, for example, about “*sensationalist*” advertising, many noted a need for advertising, but preferred advertisements to be informative.

*“…once it falls out of the prescription only category … there’s not an adequate way of having the consumer realize these products exist and are available. If the pharmacist fulfils their full professional responsibility in its sale, then there shouldn’t be a problem…”*

*Pharmacy participant*

The change from the NDPSC to the ACMS in mid-2010 did not appear to change the advertising stance (Fig. 3), despite some positive evaluator recommendations, returning to the theme of lack of trust:

*“There is a distrust that the pharmaceutical industry would advertise responsibly.”*

*Committee member*

Transparency and lack of market exclusivity allow immediate competition by manufacturers of generic equivalents, compounded by the inability to advertise:

*“…the pharmacist would recommend the in-house [generic] brand over the branded products [whose manufacturer] does all the training, provision of information, education…”*

*Industry participant*

Financial effects also influenced consumers, with some pharmacy participants noting that pensioners with time for doctor visits and low co-payments (AUS$5.80 per item concession in 2012 [€4.00]) would prefer not to pay for non-prescription products. A high co-payment (AUS$35.60 per item in 2012 [€24.80]) by consumers without a concession card was not raised as enabling switch.

#### Global or regional effects

A participant suggested companies would only launch in Australia to aid switch elsewhere. Certainly, the Australian switch attempts of orlistat (for weight loss), sumatriptan (for migraines) and montelukast (for hayfever) were early, although the last two were rejected.

Global effects such as company mergers, OTC structure and politics between the prescription and non-prescription divisions were also said to affect Australian switches.

Although participants suggested attempting switch in NZ first to try to influence Australia, this influence seemed minor, and last occurred in 2009. During 2000–2012, one of 15 progressive switches in NZ and one of nine in Australia directly arose from TTH. Third-party switches were exemption to prescription rather than scheduling changes *per se*, so were not considered in Australia under TTH. One committee member noted limited influence from NZ owing to the small market size.

#### The pharmacist-only schedule and role of pharmacy

Although participants supported having a pharmacist-only schedule and considered it facilitated switch, its benefit was moderated by advertising restrictions and sales of generics instead of the applicant’s branded product. Additionally, a regulator participant observed:

*“…S3 [pharmacist-only] is an enabler, but … it has its limitations, because if you stuck everything in S3, it wouldn’t work.”*

A dichotomy appeared around pharmacy even from the same participants. Many participants emphasized pharmacists’ cautious approach, good training, and the Quality Care Pharmacy Program. The medical participant volunteered that pharmacists are generally “*quite conservative*”. However, this participant also espoused that pharmacists cannot examine a patient, take a history, order pathologies, or diagnose. In contrast, pharmacy academics believed pharmacists did diagnose minor ailments. Various participants voiced concerns about privacy, and pharmacy behavior sometimes being substandard (including concerns about pharmacy assistants and pharmacist workload) and the committee sometimes worried about pharmacy’s role.

*“Unfortunately, in reality, it’s not always the pharmacist that you get to speak to, it’s often the 16 year old pharmacy assistant…”*

*Committee member*

## Discussion

Ours is the first research to comprehensively compare two similar countries in factors affecting switch of medicines. Participants in both countries identified some common barriers (small markets, transparency, and no market exclusivity), and enablers (progressive individuals and the pharmacist-only classification), and some differences.

Aronson[11] and Gilbert and others[7] thought that the pharmacist-only category may enable non-prescription availability, but the US Government Accountability Office was less convinced.[8] Our research supports that the pharmacist-only category is enabling, particularly with NZ’s flexible approach including exemption to prescription and mandatory pharmacist training. Gauld and others [29] described how NZ’s flexibility and pharmacist-only supply enabled calcipotriol to switch.

Current differences between Australia and NZ were suggested to reflect changes in committee membership and increasing risk concerns (Australia) and flexibility, proactivity, and third-party switch (NZ). The committees differ in composition: most members are public servants in Australia (suggested to be potentially influenced by political forces) *versus* primarily health professionals (albeit chaired by the regulator) in NZ. ‘Patch protection’ could arise from health professional organizations nominating MCC members.

No participants suggested NZ was too liberal. NZ rejected some switches,[1] and, like NZ, the UK, Germany and Sweden, has reclassified sumatriptan.[33] In 2014, the Associate Minister of Health in NZ[34] and the Chair of the MCC[35] were both positive about switch in NZ, and a NZ Medical Association spokesperson stated that the balance of medications available without a prescription was “*reasonably right*”.[36] In contrast, the Australian Medical Association has publicly opposed reclassifications,[37,38] and stated that pharmacists are not trained to diagnose, and that general practice nurses should treat minor ailments instead.[39] Duckett [40] opined that Australia had doctor-centered care with a paternalistic focus.

Some perceived differences appeared between the two countries in trust in consumers and pharmacy. We found no clear evidence of important differences between the countries in pharmacy or consumers. While Australia has a quality program that most community pharmacies have signed up to and which shows many pharmacies have provided good care,[41] other events and perhaps individual perspectives have seemingly outweighed the gains. One little study found small similarities and differences in behavior between pharmacies in NZ and Queensland, Australia.[42] Pharmacy variability in mystery shopping was evident in both countries.[43,44,45,46,47,48] The last robust covert shopping study on reclassified medicines in NZ reported reasonably positive findings,[49] while more recent studies in Australia have continued to show variability between pharmacies. Although *ad hoc* mystery shopping in NZ found all six pharmacies refused inappropriate requests for orlistat,[44] in contrast to the Australian experience,[50] limited conclusions can be drawn given the methodology and small numbers. Research on oseltamivir in NZ suggested pharmacists took their responsibilities seriously[51] and pre-switch concerns did not appear to eventuate.[52] Trust in NZ may arise from a cultural perspective; the Organisation for Economic Co-operation and Development (OECD) described NZ as *“…an open society which engenders trust*.”[53] Attitudes to risk may be similar between NZ and Australia,[54,55] but NZ is more open and liberal economically than Australia.[56] As both committees varied over the period, a strong national influence seems unlikely. Lack of trust in Australia was possibly more indicative of increasing risk averseness in Australia and media coverage of events in Australia than poorer behavior than NZ. Distrust increases both perceptions of risk and unacceptability of risk.[57]

Third-party switch (driven by neither sponsor nor government) accounted for the most recent differences between the countries, occurring often in NZ but not Australia. However, much of this took place after the interviews were conducted. We are unaware of pharmacy retailer-driven switch occurring elsewhere.

The UK has switched medicines that Australia and NZ has not.[1] Participants identified important barriers to switch in both countries. If self-care becomes policy to help widen consumer access and reduce pressure on health resources, market exclusivity and advertising (in Australia) could help. In Australia, our findings suggest that confidence in pharmacy and industry might need addressing.

Research post-switch is lacking internationally, and possibly contributes to the differing judgments reported here. Such research could inform committees in both countries, but would be costly, so it could be incentivized with market exclusivity, as in Japan.

Some participants’ views on the Australian committee resemble commentary about US advisory committee processes by Brass and Hiatt.[58] These authors expressed concerns about US advisory committee processes for medicines, e.g. high workload, inadequate member preparation, insufficient expertise, and not being evidence-based. They suggested carefully selecting committee members, correcting misinformation during the meeting, and feedback and training for members. After reviewing transcripts of US committee meetings for switches, Nguyen, *et al*.[59] recommended member training and structured committee processes. We suggest that a committee of carefully selected experts, with critical appraisal skills and good process, including training, is likely to aid robust decision-making. Literature on the dynamics in health-related committees is limited, but others have shown that individuals can affect groups[60,61] and ‘groupthink’ can affect judgment, heighten biases and sacrifice quality decision making.[62,63] Experience affects decision making[61,64] as alluded to by participants, and NZ’s positive experience with oseltamivir, and Australia’s negative experience with orlistat may have influenced later decisions.

### Limitations and Strengths

Participant recall and biases can affect interview findings. Meeting records are also inherently limited, being a summary, and tension, individual dynamics and performance are not documented. Meeting records can sometimes also be inaccurate.[65] We combined interviews and meeting records to minimize these limitations.

Two participants sometimes represented the same organization, or the same role (e.g. two pharmacy academics in Australia, or different committee members). Such interviews differed, partly from the semi-structured approach, but also reflecting different involvement and experiences in switch. More interviews might add information, but the number of people involved in switch is limited, and we interviewed the most appropriate person(s) from each organization. Given the wide and long-standing experience of switch from most of our participants, and the long interviews, major barriers and enablers should have arisen for both countries. Adding less-informed participants probably would have provided little extra information and potentially more speculation. Other stakeholders, such as academics in general practice, practicing community pharmacists or health policy experts, could add different viewpoints. However, they may have little experience of reclassification and we sought participants with the most experience,[28] but also including medical and consumer views. We included the key people involved in reclassification from regulators, pharmacy organizations, industry and committees considering switch. Achanta, *et al*.[12] interviewed 18 key opinion leaders from four countries, and used statements from another 18 key opinion leaders in considering how to improve US regulatory processes for non-prescription medicines, including switch. Whether any of their participants were switch committee members was unclear, but participant experience included medicines regulator work, academia, industry and consumer organization work.

The timing of interviews would have affected content. Most interviews were conducted in 2010 and 2011. Thus, little recent NZ pharmacy-retailer driven switch activity was reflected in the interviews. Australian interviews preceded extensive experience with the new ACMS, but some concerns expressed about the NDPSC composition may continue with the jurisdictional member majority remaining. Advertising and switch approval data do not indicate the ACMS has become more open to switch than the NDPSC was just before the change.

The heuristic approach uses the knowledge, experiences and insights of the lead researcher.[27] Her long-standing involvement in NZ switches from multiple perspectives and awareness of switch in Australia and attendance at an NDPSC meeting facilitated the research, but has potential for bias. Use of documents for triangulation, quotations, participant reviews of country reports, and skeptical peer review helped to limit the bias. A different interviewer might have elicited some different findings, although many of our findings (e.g. conservatism in Australia and evidence-based decisions and flexibility in NZ) came from multiple participants, were supported by meeting records, or both. Participants were selected based on their direct involvement in switch (e.g. consumer and medical voices, Presidents of pharmacy organizations, industry personnel most involved in switch) and not because they were known to NG or known to share her views. The interviewer’s relationship with some participants might have affected their responses, possibly providing a level of trust, encouraging sharing, or shaping answers to please the interviewer. However, participants were highly experienced, senior people, most of whom were not well known to NG, and NG did not share her views until late in the interview to help mitigate this possibility. Participants checking the country reports did not indicate significant concerns.

Further research could investigate realized benefits and risks of switch in Australasia to understand consequences of the differences seen. Future research could include interviews of personnel within the States and Territories who influence switch, and explore the influence of the change from the NDPSC to the ACMS. We recommend further research into optimizing committee performance, including the ideal committee constitution for considering switch. Observing meetings and interviewing all committee members may add insight into committee dynamics and differences between the countries. Observing different committees considering switching the same medicine may provide useful insight.

## Conclusions

Multiple reasons appear to lie behind differences in medicines switch (and hence potential for consumer self-care) between two similar neighboring countries; awareness of these may shape future harmonization policy implementation. Reasons include suggestions of increasing conservatism and political influences in Australia, and flexibility and the rise of ‘third-party switch’ in NZ. Pharmacist-only scheduling appears to aid switch, particularly where a flexible approach is used, but barriers to switch, including perceptions of pharmacy and consumer behavior, lack of market exclusivity and small market size, can moderate such effects.

Consumers and the health system in NZ may be benefiting from its environment enabling switch, which allows more opportunities in self-care or shared care than consumers have in Australia.

## Supporting Information

S1 Appendix Flowchart of research. (TIFF)
