# Supplementary figures and images for: Widening Consumer Access to Medicines: A Comparison of Prescription to Non-Prescription Medicine Switch in Australia and New Zealand
Source: PLoS One. 2015 Mar 18;10(3):e0119011. doi: 10.1371/journal.pone.0119011 (PMC4364766; doi:10.1371/journal.pone.0119011)

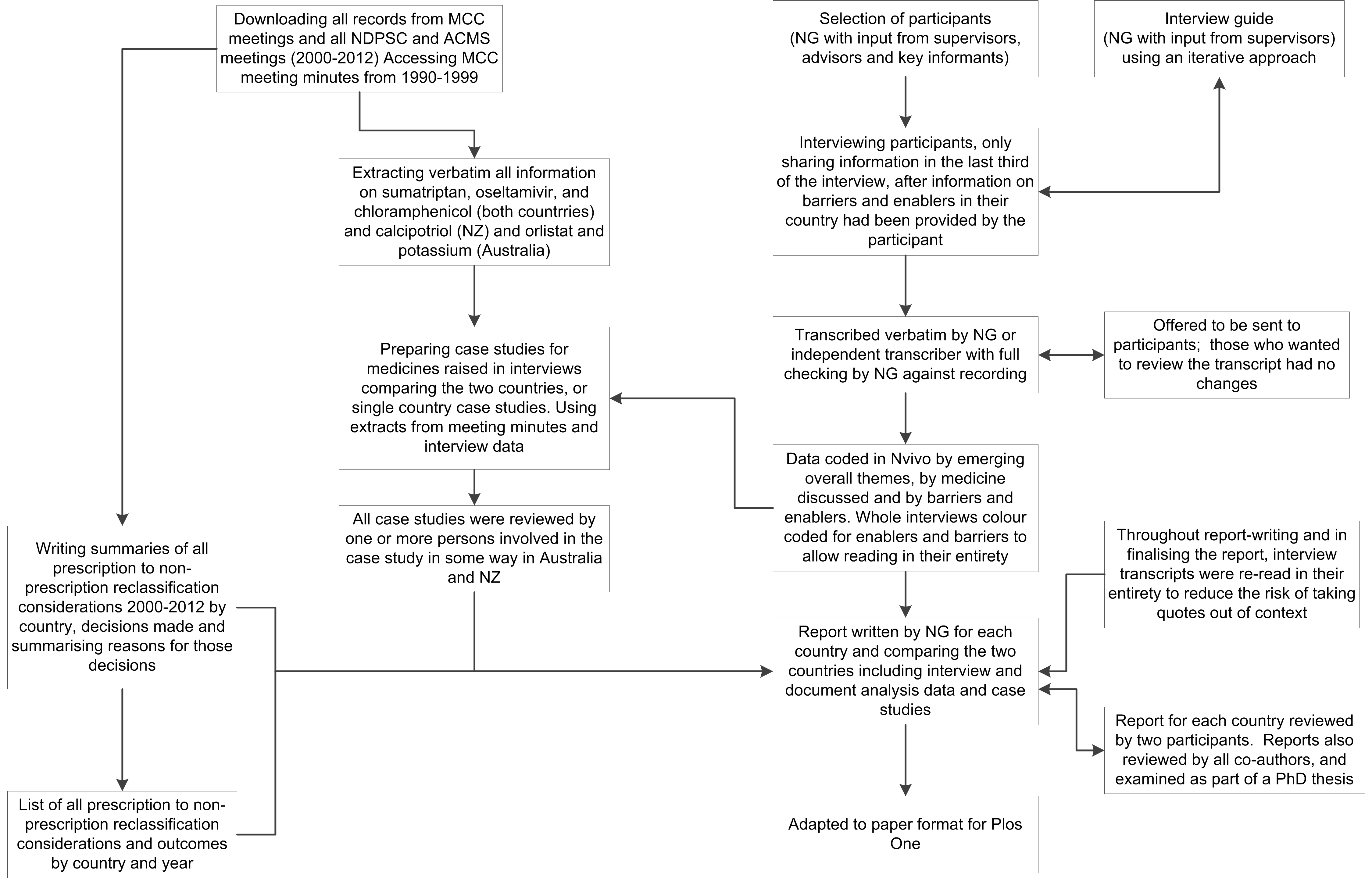

Supplement: S1 Appendix — (TIFF) [file pone.0119011.s001.tiff]
